# Systematic Review of Self-Assessment in Physical Education

**DOI:** 10.3390/ijerph18020766

**Published:** 2021-01-18

**Authors:** Fernando M. Otero-Saborido, Victor Torreblanca-Martínez, José Antonio González-Jurado

**Affiliations:** School of Sport Sciences, Pablo de Olavide Universtiy, 41013 Sevilla, Spain; victm81@hotmail.com (V.T.-M.); jagonjur@upo.es (J.A.G.-J.)

**Keywords:** self-assessment, formative and shared assessment, self-regulation, metacognition

## Abstract

Self-assessment is among the most impactful processes in student learning. Since no review of this process was found in the field of physical education (PE), the objective of this work was to perform a systematic review of the literature published over the last five years on the self-assessment of PE students in formal education contexts. The review was conducted in accordance with the Preferred Reporting Items for Systematic Reviews and Meta-Analyses (PRISMA) standards. Inclusion and exclusion criteria were established to select the articles. A total of three researchers independently applied the above criteria and obtained a total of thirteen studies. After synthesising the results by category, we found that: a majority of the studies were conducted in Europe; all 13 studies covered the educational stages of either secondary or higher education; an even number of qualitative and quantitative approaches were distributed among the studies; some studies focused on specific sports or contents, while others were applicable to any subject; and, finally, depending on the research design adopted, the results described self-assessment strategy processes, improvements in learning, drew descriptive portraits of students regarding health, or reflected students’ positive perceptions of self-assessment. It is necessary to conduct further studies on PE students’ self-assessment, especially in primary education.

## 1. Introduction

### 1.1. Training and Shared Assessment in Education

Assessment cannot be considered as a mere technical process but as an action with ethical, ideological, and political connotations [1]. Therefore, depending on the purposes assigned to the assessment, these connotations acquire one meaning or another. It is not the same to assess in order to rate academic performance, control, punish, or supervise students than to assess as a means to verify the teaching–learning conditions to motivate students or to foster the inclusion of learners regardless of their abilities. The objective of the present study was to conduct a systematic review concerning this latter meaning of assessment, also referred to as formative and shared assessment [2]. The work by Black and William [3] may be regarded as the first study that gathered evidence on the benefits of formative and shared learning assessment. Nevertheless, despite the methodological limitations of Black and William’s study pointed out by some authors, there is another aspect brought about by formative and shared assessment that is worthy of mention beyond technical improvement: the educational and social nature of assessment as an inclusive process. The definition given by Alcaraz [4] seems accurate: “We claim that we are assessing students in order to check whether students are learning or not, but we are forgetting that the main function of assessment is less that of verifying the learning and more to ensure that the conditions for such learning to occur are being met.”

Therefore, as pointed out by López-Pastor and Pérez-Pueyo [2], we understand formative assessment as any process “whose main objective is to improve the teaching–learning processes that are taking place. It helps students to learn more (and/or correct their mistakes) and teachers to work better (to improve their teaching practice).” In this sense, Spain’s Formative and Shared Assessment Network in Education (REFyC) added the qualifier “shared” because of the critical role of the active participation of the different agents in the teaching–learning processes. In this line, scientific evidence is highly consistent regarding the positive impact of feedback on learning progress. A meta-analysis carried out by Lyster and Saito [5] verified the efficiency of oral feedback on improving one’s learning (with an average effect size of 0.76), especially when it was sustained over time. Other studies have demonstrated such influence in terms of the type of feedback to be used and its frequency, invariably leading to notably beneficial results for students [6,7,8]. The different degrees to which students take part in their own assessment were classified into several categories: self-assessment, coassessment, shared assessment, qualified dialogue, and self-qualification [9]. Self-assessment, the object of the present research review, is nothing more than an individual’s own assessment of a process and/or result. 

### 1.2. Self-Assessment as a Formative and Shared Assessment Process in Physical Education

In the case of physical education (PE), the subject addressed in the present systematic review, there are two very different assessment models. The first model is oriented towards physical performance and seeks to measure the effectiveness of the students’ physical performance through exams and standardised tests. This conception does not contemplate the assessment’s formative value. Its intention is well summarised in the question expressed by López-Pastor [10]: “Why do we say assessment when what we really mean is qualification?” In this paradigm, students assume a passive role in the teaching–learning process. As opposed to the former, the student-participation model does have a high educational value. It focuses not only on the motor aspect but also contemplates the affective, social, and cognitive dimensions by involving students in the assessment. Furthermore, participation strategies such as self-assessment, dialogue qualification, or coassessment are possible in this evaluative model. In the case of PE, different studies have demonstrated the benefits of involving students by using self-assessment situations as well as peer assessment or coassessment [11,12,13,14,15]. Reviews of different works on assessment practices over a range of contexts and points in time in PE reveal that despite the prevalence of the formative assessment paradigm, grading models continue to predominate in PE teachers’ evaluative practices, with low student participation [16,17,18].

Moreover, the strategy of self-assessment as a student participation process within shared and formative assessment is closely linked to learning self-regulation. The concept of learning self-regulation involves a number of cognitive processes that fall into four big categories: metacognition, self-control, emotional self-regulation, and resilience [19]. Self-assessment is part of each. In the case of metacognition, for students to learn to learn, they must first evaluate their point of departure, and to do this, they must resort to self-assessment. The same applies to self-control and emotional self-regulation, where students learn to activate inhibitory control as they gain knowledge of their own emotions by evaluating themselves. We may ask: what effect do these self-assessment skills have on learning? The review by Bartimote-Aufflick et al. [20] revealed a highly consistent and strong association between self-efficacy and student learning outcomes. In the same way, in the 64 studies included in the review, self-efficacy was linked to processes that were directly linked to assessment such as self-control, intrinsic motivation, or self-regulation. In the case of PE, although some studies were directly related to some of these processes [15,21,22,23,24,25,26,27], we did not find a systematic review on students’ self-assessment that outlined these processes in PE classes in formal educational contexts. Therefore, the objective of this work was to conduct a systematic review of the literature over the last five years on PE students’ self-assessment in formal education contexts. 

## 2. Materials and Methods

### 2.1. Inclusion Criteria 

The search keywords were “physical education” and “self-assessment.” Both were used as inclusion criteria in the review using the AND command within the fields “article,” “keywords,” and “abstract.” The period covered was limited to the last five years, between 2016 and 2020. In this sense, the term self-assessment was contextualised within the definition offered by the REFyC: “Assessments performed by people themselves on their own processes and/or results. Self-assessment may be performed by students or teachers. It can also refer to personal or group self-assessment” [2].

There are different types of reviews on a topic: narrative review, systematic review, and meta-analysis. Since the topic of this research is framed in the sciences of education, the systematic review was the selected modality. Systematic reviews represent a specific type of research in which the units of analysis are the original primary studies. The Preferred Reporting Items for Systematic Reviews and Meta-Analyses (PRISMA) guidelines [28] were followed. The PRISMA statement standards consist of a 27-item checklist spread across the Title, Abstract, Introduction, Method, Results, and Discussion sections. The PRISMA statement items were checked for each article, one by one. Item Numbers 4 and 6 that refer to the PICO format were of particular interest: “Provide an explicit statement of questions being addressed with reference to participants, interventions, comparisons, outcomes, and study design (PICOS)” [28]. In the present study, the “participants” were the “educational stage” (primary, secondary, or higher education), the “interventions” corresponded to the “method” (qualitative and quantitative approaches), and comparisons were included in “self-assessment” as search criteria.

### 2.2. Exclusion Criteria

A total of 5 exclusion criteria were defined:

1. The article was not included in the Web of Science or Scopus databases.
2. The term ‘Self-assessment’ was not contextualised in the semantics of the REFyC.
3. The research was based exclusively on the teacher’s self-assessment.
4. The research was conducted in contexts other than regulated education.
5. The article was published in a language other than English or Spanish.

### 2.3. Procedure

The study unfolded over 3 phases. The first and third phases were jointly performed by the three researchers. The second phase was carried out independently by each of them as a triple-blind study. 

#### 2.3.1. First Phase 

The three researchers practised applying the inclusion and exclusion criteria. In the case of the inclusion criteria, it was found that the term “self-assessment” was the one used by the scientific community to designate the review’s object of study. Likewise, the exclusion criteria perspective was unified, and special attention was paid to number 2 (“The term “self-assessment” was not contextualised in the semantics of the REFyC”). To unify the criteria, several examples of somewhat ambiguous cases were given. To finish, the 27 PRISMA standard items were reviewed. 

#### 2.3.2. Second Phase 

**Figure 1:**
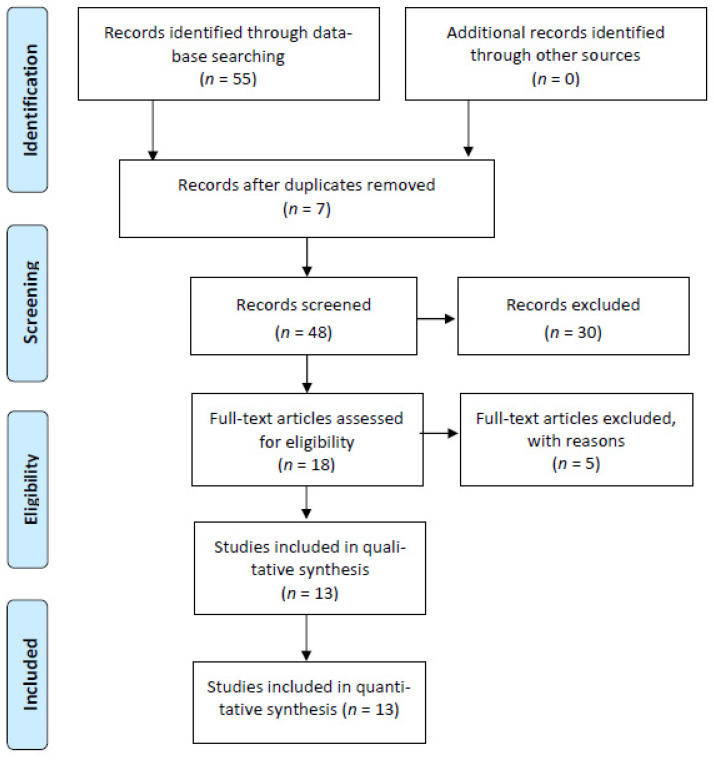
Flowchart describing phase two of the review process.

Based on the inclusion and exclusion criteria indicated above, we performed a search in three databases: Scopus, Web of Science, and ERIC. A first search produced a total result of 45 articles across the three databases (Figure 1). All works had been published between 2016 and 2020. Subsequently, after applying the exclusion criteria from 1 to 5, the number of studies dropped to 29, distributed across each database, as illustrated in Figure 1. The researchers eliminated the resulting duplicate articles after applying the inclusion and exclusion criteria. Finally, the PRISMA standards were verified, taking into account that the object of study mainly focused on educational sciences.

#### 2.3.3. Third Phase

The three researchers shared the results of phase two, demonstrating a high level of agreement. Two researchers found the same final 13 studies, and a third researcher found 14. The exclusion criteria and the PRISMA standards were reapplied, the review finally resulting in 13 studies on PE students’ self-assessment in formal education contexts. An agreement was reached to organise the results into the following categories: (1) Author and year of publication; (2) Country: for this category, the location of the study was taken into account; (3) Educational stage: the educational stages addressed in the studies were taken into account or, where unspecified, were defined according to the students’ ages. Based on this criterion, the primary, secondary, and higher education stages categories were found; (4) Method and instrument: quantitative, qualitative as well as mixed model approaches were found; (5) Objectives: the objectives of the studies were explicitly included; (6) Content: this section included the type of PE-related content referred to in the study. In some cases, the content was not specified or was indifferent, so “All curricular contents” was indicated; (7) Results: the research results were synthesised. All phases were conducted between 15 September and 2 November 2020.

## 3. Results and Discussion

The seven categories listed in Table 1 (except author and year) were described and discussed in the 13 articles composed between 2016 and 2020.

### 3.1. Country

Europe, with twelve studies [27,29,30,31,32,33,34,35,36,37,38,39,40], and America [41], with one, were the only continents to be represented in the systematic review on self-assessment in physical education. In the case of Europe, Spain was the country with the most articles on the subject, with a total of five [30,33,35,37,40]. Other countries were represented on this continent such as Ukraine [38,39], Belgium [36], France [34], Norway [29], and Portugal [32]. The reason other continents were nonrepresented is that many articles met exclusion criteria such as self-assessments carried out in sport contexts (not educational ones) or referred to the self-assessment of PE teachers instead of students. Moreover, the high number of articles in Spain is due to the influence of the Shared and Formative Assessment Network led by López-Pastor [12] (University of Valladolid, Spain). This network has been investigating the processes of formative and shared assessment in physical education for over 30 years.

### 3.2. Educational Stage

In this category, it is worth noting that secondary and higher education (with seven and six studies, respectively) were the only educational stages addressed in articles on self-assessment in physical education. In the case of Spain, four [30,33,35,40] of its five articles focused on a university setting and only one [37] on secondary education. The two articles from Ukraine were also directed towards university teaching. The other articles centred on secondary education were distributed across Norway, Portugal, Chile, Belgium, and the United Kingdom. The absence of studies on self-assessment in physical education in the primary stage was conspicuous in this systematic review. These results do not coincide with the recent review conducted by Bores-García, Hortigüela-Alcalá González-Calvo, and Barba-Martín [42] on coassessment in physical education. In that study, the primary stage was represented by coassessment formative assessment strategies. In this sense, it could be interpreted that the self-assessment object of this review is a more appropriate cognitive process for stages with greater cognitive maturation. However, the excessive standardisation of primary education curricula in physical education hinders the existence of formative assessment practices. This conclusion is drawn from the studies by Otero-Saborido, Vázquez-Ramos, Cenizo-Benjumea, and González-Jurado [43,44] on PE curricula. These curricular models are based on the measurement of quantifiable behaviours through standards. For this reason, formative and shared assessment (self-assessment and coassessment) takes second place with respect to heteroassessment practices (teacher–student) oriented towards qualification.

### 3.3. Methods and Type of Research

The studies were heterogeneous in terms of design. Five papers exclusively used qualitative research [29,30,31,33,37]. Of these five studies, the works by Aarskon [29] and Macken, MacPhail, and Calderon [31] are worthy of mention regarding the wide range of qualitative instruments used. In the case of the first, participant observation, interviews, and video analysis were applied to collect students’ reflections on their PE learning processes. In the case of the second, the design was action research, where field notes, postlesson debriefing, reflective journals, semistructured interviews, and focus group interviews were used. In addition, these qualitative studies all shared the same object of research, i.e., the learning process and not its result.

A similar amount of works adopted a qualitative research approach, i.e., four articles [32,34,36,38]. In all these works, the instrument was a structured questionnaire. These quantitative studies shared a common understanding that PE self-assessment is the reflection that students make on the final result of their learning or physical activity habits, and not on the process leading up to those results. This type of “result-oriented” self-assessment overlooks the process and involves students in a testimonial way. For this reason, it may not always lead to improved student performance but rather serve the researchers’ descriptive objectives. This is the case of the study by De Meester et al [36], where 216 adolescents completed questionnaires on PA, sports participation, motivation for PE, and perceived motor competence, undertaking a series of tests to assess their actual motor competence.

Finally, out of the 13 studies, four used a mixed methodology that combined qualitative and quantitative instruments [35,39,40,45]. A general assessment of the type of research used shows that there was no traditional dominance of the positivist model based on quantitative instruments. In the present review, a balanced ratio was found between qualitative and quantitative paradigms. 

### 3.4. Purpose and Content

Two big categories emerged: on the one hand, studies that did not focus on specific content or a specific objective, and on the other, studies that did focus on specific content and sports. In the case of the former, two subgroups could be defined: a first subgroup that focused on the learning process, without explicitly describing or detailing the content object of self-assessment [29,31]. This was the case of the study by Aarskon [29], that aimed “to explore how students themselves participate in the assessment processes that occur in PE.” Although floorball is mentioned on several occasions in this study, the text is narrated as if it were applicable to any content. Within this group of studies that did not focus on specific content or a specific objective, a second subgroup was found, which centred self-assessment on very general aspects of healthy habits, motor competence, motivation in PE classes, and specific populations [36,38,41]. This was the case of the study by Griban et al. [38], which aimed “To analyse the factors that affect the students’ health both positively and negatively and to evaluate the real health status of Ukrainian student youth.” In this latter work, the students assessed themselves by filling out questionnaires on aspects such as the weekly frequency with which they performed physical activity, drug use, or hours of sleep. Within the subgroup that did not specify the content, other examples include the research by De Meester et al. [36], in which the students’ self-assessment was not limited to sports or specific physical activities since the objective was “to explore how motor competence-based profiles relate to motivation for PE, PA levels, and organised sports participation.” Finally, another study worthy of mention is that of Marques et al. [32], which addressed the physical activity habits of students with spina bifida.

A second large group of studies addressed very specific content and sports. Two studies on gymnastics ([33,34] and corporal expression [30,35] were found, respectively. One study aimed “To design, implement and observe the motor response to a volleyball didactic unit based on nonlinear pedagogy principles” [37], and another dealt with aspects related to physical condition and health but in a very specific way. This was the case of the study by Hakman et al. [39], which sought “To determine the increase in indicators of physical qualities and circumference sizes of the body in students as a motivational component of physical self-improvement.”

### 3.5. Outcomes

Although all studies included self-assessment, the methodological approaches used and the objectives of the studies conditioned their results. A first group of studies used questionnaires as their main instrument and the self-assessment consisted of filling out a questionnaire on health habits and physical activity, the results of which consisted of drawing demographic profiles of physical activity and health, motivation, or motor competence of the sample. Examples include the studies by Griban et al., Marques et al., and Meester [32,36,38]. For example, in the case of Griban et al. [38], the students stated that the health factors they considered to be the most dangerous included drug use, radioactive contamination of the environment, smoking, alcohol abuse, stress, etc.

Other studies presented results that could be included within quasiexperimental studies or correlations. This type of research aims to determine how self-assessment influences or leads to learning progression. This was the case of the study by Potdevin et al. [34], which, based on a control and experimental group, evaluated the influence of self-assessment using video feedback on gymnastic skills and motivational profiles. The results showed statistically significant improvements in the experimental group in their execution of gymnastic skills. In the case of the study by Gómez-Criado and Valverde-Esteve [37], a high significant correlation was found between knowledge of the game and self-perception (self-assessment) of the competence of different technical skills in volleyball such as passing, receiving, the serve, the block, or the attack.

Moreover, a third group of studies described the entire process of student involvement or student perceptions without seeking to achieve statistical generalisations based on cause–effect relationships. This was the case of studies such as those by Aarskon [29] or Macken, MacPhail, and Calderon [31]. In the latter case, the results showed that the use of teacher educator modelling, mentoring, and scaffolding with primary school students, during upskill sessions and in situ during preservice teachers’ (PST) school placements, enhanced the primary preservice teachers’ (PST) assessment literacy in the enactment of assessment for learning in primary physical education to a greater extent than when implemented during the module with their PST peers. In the study by Romero-Martín and Asún, Chivite-Izco [35], physical education university students showed their satisfaction regarding the self-assessment system used in the Body Expression subject. A similar conclusion was reached by López-Pastor [40], who found that university students who performed self-assessment practices valued how the assessment criteria’s clarity and transparency made a very positive contribution to the teaching–learning process.

After synthesising the results we found that: a majority of the studies were conducted in Europe; all 13 studies covered the educational stages of either secondary or higher education; an even number of qualitative and quantitative approaches were distributed among the studies; some studies focused on specific sports or contents, while others were applicable to any subject; and finally, depending on the research design adopted, the results described self-assessment strategy processes, improvements in learning, drew descriptive portraits of students regarding health, or reflected students’ positive perceptions of self-assessment.

## 4. Conclusions

The main contribution of this research is that it is the first systematic review found to date on self-evaluation in physical education. At first, a large number of studies were found, but after applying the exclusion criteria, the number of works was reduced to 13. Some were excluded because the meaning of the word “self-assessment” was interpreted out of the educational context assumed in this research. Others were discarded because the studies were conducted in noneducational contexts (sometimes exclusively in the field of sport). The final results reflected that the number of studies on self-assessment in PE was very small and geographically limited to the European continent. University and secondary education were addressed in the studies to the detriment of primary education, where no works were found that did not meet the exclusion criteria. A numerical balance between quantitative and qualitative methods was found with respect to the research and/or applied self-assessment processes in PE. Nevertheless, although qualitative studies seek to reach a holistic understanding of the use of self-assessment in PE, in some quantitative studies, students’ self-assessments served descriptive purposes that did not necessarily improve students’ teaching–learning processes. Other quantitative studies seemed to show that the use of self-assessment improves students’ performance in the teaching–learning process. Students’ perceptions were generally highly positive about the use of self-assessment in PE. Further studies on PE self-assessment in educational contexts should be performed, particularly in primary education, for which no research was found.

## Figures and Tables

**Table 1:** Summary of articles about self-assessment in physical education (PE) published between 2016 and 2020.

Author and Year	Country	Educational Stage	Methods and Instrument	Purpose	Content	Outcomes
Aarskog, G. (2020)	Norway	Secondary	Qualitative approach. Participatory observations, video analysis and interviews.	To explore how students themselves participate in PE assessment processes.	All curricular contents	The findings were presented according to these three questions: where are the learners in their learning, where are the learners going in their learning, and how will they get to where they are going?
Asún Dieste, S., Rosario Romero-Martín, Ma., Aparicio-Herguedas, J.L., Fraile-Aranda, A. (2020)	Spain	Higher Education	Qualitative approach. Self-report and content analysis.	To ascertain preservice PE teacher training students’ perceptions of their teaching competence in terms of proxemic nonverbal communication.	Body expression	The students underscored the importance of both physical and emotional immediacy to create an optimal teaching and learning space.
Ovalos, M.A., Garde, A., Vega, L. (2020)	Spain	Secondary	Qualitative approach. Semistructured interview and content analysis.	To analyse students’ perceptions after carrying out an acrobatic gymnastics didactic unit using the technologies and involving the students in the assessment process.	Acrobatic gymnastics	The use of video and the inclusion of the students in the assessment allowed them to take part in their teaching–learning process, contributing to the training of more critical and autonomous people.
De Meester, A., Maes, J., Stodden, D., Cardon, G., Goodway, J., Lenoir, M., Haerens, L. (2016)	Belgium	Secondary	Quantitative approach. Questionnaire	To explore how motor competence-based profiles related to motivation for Physical Education, Physcial Activities levels, and participation in organised sports.	All curricular contents	After the students’ assessment, the results emphasised that developing perceived motor competence (MC), especially among adolescents with low levels of actual MC, seemed critical to stimulate motivation for PE and engagement in PA and sports.
Flores Bernal, R.F., Pérez, A.M., Martínez, P.J., Queipo, E.A.B., Zamora, B.M. (2019)	Chile	Secondary	Quantitative and qualitative approach. Questionnaire.	To analyse the teaching and methodological factors affecting the scant motivation of eighth-grade female junior high school students to participate in physical education classes.	All curricular contents	The analysis confirmed that eighth-grade female students lacked motivation and did not attend physical education class regularly since those activities had little in common with adolescents’ interests and likings.
Gómez-Criado, C., Valverde-Esteve, T. (2021)	Spain	Secondary	Qualitative approach. Rubrics.	To design, implement, and observe the motor response to a volleyball didactic unit based on nonlinear pedagogy principles, such as the modification of the rules, size of the game area, or material used.	Volleyball	There was a high significant correlation between the knowledge of the game and the self-perception competence of the pass (r = 0.366, p = 0.004), reception (r = 0.266, p = 0.040), serve (r = 0.376, p = 0.003), body collocation (r = 0.413, p = 0.001), and attack and block (r = 0.267, p = 0.038).
Griban, G.P., et al., (2019)	Ukraine	Higher Education	Quantitative approach. Questionnaire.	To analyse the factors that affect the students’ health, both positively and negatively, and to evaluate the real health status of Ukrainian students’ youth.	All curricular contents	The students stated that the most dangerous health factors included drug use, radioactive contamination of the environment, smoking, alcohol abuse, stress, etc. The students’ self-assessment of their health state allows managing the educational process of physical education efficiently, allocating physical activity, and applying individual tasks rationally.
Hakman, A., et al,. (2020)	Ukraine	Higher Education	Quantitative and qualitative approach. Portfolio.	To determine the increase in indicators of physical qualities and body circumference sizes in students as a motivational component of physical self-improvement	Health and physical condition	The dynamics of deterioration of physical fitness starting from the second year, as identified by the assessment, determined the selection of second-year students for the introduction of the developed “individual portfolio for the self-control of students’ physical condition,” which involved self-management and self-control over students’ own physical condition, with the teacher being responsible for the informational and corrective aspects.
López-Pastor, V.M., Pérez-Pueyo, Barba, J.J., Lorente-Catalán, E. (2016)	Spain	Higher Education	Qualitative and quantitative approach. Semistructured interview and questionnaire.	To identify the perception of university students on Preservice Teacher Education (PTE) courses on the importance and functionality of assessment criteria rubrics when undertaking written group assignments.	All curricular contents	(a) Prior knowledge of the grading criteria helped them to perform the task better; (b) significant differences were found among the students’ previous experiences of this type of assessment instrument, depending on the university where they studied; (c) students felt that using a rubric (self-assessment and peer assessment) while undertaking a group assignment was very valuable.
Macken, S., MacPhail, A., Calderon, A. (2020)	Ireland	Higher Education	Qualitative approach. Action-research. Field notes, postlesson debrief, reflective journal, semistructured interviews, and focus group interview	To examine the extent to which primary preservice teachers (PTs) demonstrate assessment literacy in their enactment of assessment for learning (AfL) while teaching physical education during school placement.	All curricular contents	The use of teacher educator modelling, mentoring, and scaffolding with primary school students, during upskill sessions and during the PST school placements, enhanced the PSTs’ assessment literacy in the enactment of AfL in primary physical education to a greater extent than when implemented during the module with their PST peers.
Marques, A., Maldonado, I., Peralta, M., Santos, S. (2015)	Portugal	Secondary	Quantitative approach. Questionnaire.	To identify psychosocial correlates of physical activity among children and adolescents with spina bifida.	Health	Most of the children and adolescents did not participate in physical activity regularly, and sociodemographic and psychosocial variables were not related to organised and nonorganised physical activity, except perception of competence.
Potdevin, F., Vors, O., Huchez, A., Lamour, M., Davids, K., Schnitzler, C. (2018)	France	Secondary	Quantitative approach. Questionnaire and arm–trunk angle progression.	To assess the effects of a methodology combining video feedback, attentional information and verbal instructional constraints on the learning of a gymnastic skill, motivation during learning, and students’ self-assessment ability.	Gymnastics.	The results of this study showed how using a simplified VFB-based learning aid, coupled with a self-assessment task, in real-life teaching conditions during an ongoing PE programme contributed to enhancing motor skills, self-assessment ability, and motivation profiles over a short period of time in novices.
Romero-Martín, M.R., Asún Dieste, S., Chivite-Izco, M.T. (2016)	Spain	Higher School	Qualitative and quantitative approach. Semistructured interview and questionnaire.	To know the perceptions of students and graduates on good practice of formative assessment in body language.	Body expressions	The results suggest that this proposal of good practice was highly valued by students. The students found the experience innovative; the students highlighted the value of teacher and peer academic support.
